# Introns and gene expression: Cellular constraints, transcriptional regulation, and evolutionary consequences

**DOI:** 10.1002/bies.201400138

**Published:** 2014-11-14

**Authors:** Patricia Heyn, Alex T Kalinka, Pavel Tomancak, Karla M Neugebauer

**Affiliations:** 1) MRC Human Genetics Unit IGMM, University of EdinburghEdinburgh, UK; 2) Institute of Population Genetics, Vetmeduni ViennaVienna, Austria; 3) Max Planck Institute of Molecular Cell Biology and GeneticsDresden, Germany; 4) Molecular Biophysics and Biochemistry, Yale UniversityNew Haven, CT, USA

**Keywords:** cell cycle constraints, gene length, macro-evolutionary patterns, splicing

## Abstract

A gene's “expression profile” denotes the number of transcripts present relative to all other transcripts. The overall rate of transcript production is determined by transcription and RNA processing rates. While the speed of elongating RNA polymerase II has been characterized for many different genes and organisms, gene-architectural features – primarily the number and length of exons and introns – have recently emerged as important regulatory players. Several new studies indicate that rapidly cycling cells constrain gene-architecture toward short genes with a few introns, allowing efficient expression during short cell cycles. In contrast, longer genes with long introns exhibit delayed expression, which can serve as timing mechanisms for patterning processes. These findings indicate that cell cycle constraints drive the evolution of gene-architecture and shape the transcriptome of a given cell type. Furthermore, a tendency for short genes to be evolutionarily young hints at links between cellular constraints and the evolution of animal ontogeny.

## Introduction

According to the central dogma, DNA makes RNA and RNA makes protein. The important functional unit within DNA is the gene, which is transcribed by RNA polymerase and templates either protein-coding or non-coding RNA. Approximately 10% of the eukaryotic genome is comprised of genes, while 90% is intergenic [1]. It is the job of regulatory DNA-binding proteins, transcription factors, to identify genes within DNA and recruit the appropriate RNA polymerase to transcription start sites. Once transcription initiates, it remains for RNA polymerase to elongate and terminate the RNA transcript. Transcription initiation and termination are clearly one-time events per transcript. However, because genes vary dramatically in their length, transcription elongation is the part of the transcription cycle that varies on a gene-by-gene basis.

A gene's length multiplied by the average elongation rate determines how long it takes to transcribe that gene. In eukaryotes, average transcription elongation rates for RNA polymerase II (Pol II) have been determined in vivo, using a variety of techniques and yielding values from 1 to 5 kbp/min [2]. As a greater number of genes become considered, it appears that an average elongation rate of 1.5 kbp/min is generally applicable to most genes, although a trend toward more rapid elongation through long genes has been noted [3,4]. The range of these values may be, at least in part, due to the susceptibility of transcription elongation to regulation by signaling [5]. Elongation rates are also influenced by histone post-translational modifications, and higher density of exons is correlated with slower average elongation rates; the latter is possibly due to Pol II pausing over exons, in which nucleosomes can be positioned [2–4]. All this indicates that gene architecture contributes to the establishment of gene-specific transcription elongation rates that vary within an order of magnitude.

**Figure 1:**
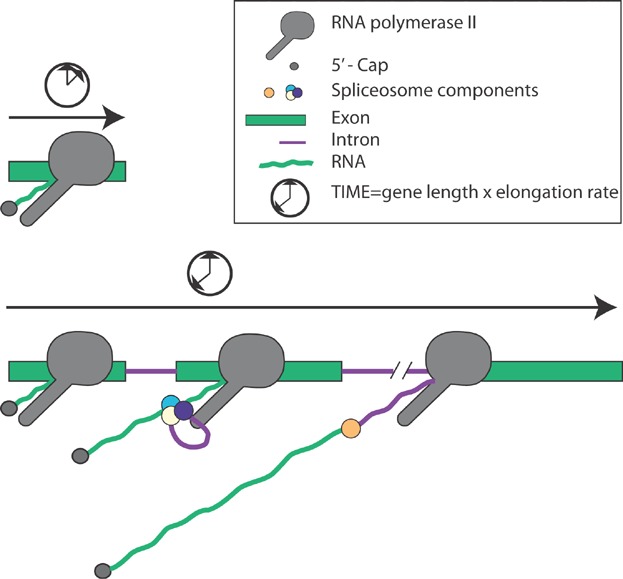
Transcription and RNA processing take time. A schematic on an intronless (upper panel) and an intron-containing gene (lower panel) are depicted. Pol II transcribes the genes and the RNA is co-transcriptionally capped at the 5′-end as well as spliced (intron-containing gene). The time it takes for Pol II to reach the end of the gene depends on the length of the gene and the elongation rate of Pol II.

Gene lengths also vary by many orders of magnitude. For example, one of the smallest human genes, U7 snRNA, is only 63 base pairs (bp) long, while the human dystrophin gene is longer than 2,000,000 bp. Among the shortest protein-coding genes are the histone genes, ∼400 bp long. One factor contributing to this size difference is the presence or absence of introns, usually non-coding parts of the gene that reside between the exons. Introns are removed from the transcript during the process of pre-mRNA splicing, which produces mature mRNA from the exons (Fig. 1) [6]. In the above examples, U7 and histone genes are among the 5% of human genes that are intronless [7]. In contrast, the longest annotated dystrophin transcript harbors 78 introns, which contribute ∼99.3% of its gene length. Transcription of the dystrophin gene takes 16 hours due to the gene's excessive length [8]. The median human gene length is 20,000 bp, which corresponds to ∼10–20 minutes of transcription time, assuming the elongation rates discussed above. In addition, the size of introns varies widely and there is a general trend for shorter introns in more basal species and longer ones in primates [9]. In contrast, there seems to be an evolutionary pressure to keep the exon length at ∼140 bp [10], approximately the length of DNA that wraps around a nucleosome. The correspondence between nucleosome size and internal exon length is strong; but this exon size may also be favored in evolution, due to exon length constraints on splicing mechanisms [11]. Nevertheless, it is clear that the time it takes to transcribe a eukaryotic gene will be heavily influenced by its gene architectural features, in particular the presence and abundance of introns.

## Can RNA processing influence gene expression rates?

Does RNA processing itself contribute a rate that impacts the overall rate of gene expression? Capping of the 5′-end as well as 3′-end cleavage and polyadenylation are rapid RNA processing events associated with singular events of transcription initiation and termination. On the other hand, in vivo splicing rates are difficult to measure directly and could be variable due to a high level of regulation. Several estimates suggest that splicing takes ∼30 seconds to 3 minutes from the time of 3′ splice site synthesis in vivo and so could impose significant overhead on the overall gene expression rate [12]. If, however, splicing were to occur exclusively during elongation, i.e. co-transcriptionally (Fig. 1), then RNA processing would not contribute to gene expression rates at all. Recent genome-wide studies have established that intron removal is mostly (∼75%) co-transcriptional from yeast to human [12,13]. Widespread co-transcriptional splicing suggests that gene expression is primarily defined by transcription time alone. Additionally, pausing within terminal exons delays the transcription of intron-containing genes, adding to total time it takes for gene expression [14]. Introns that are not efficiently removed co-transcriptionally may display more significant delays in gene expression. Examples include intron retention in the gametes of fern spores, undergoing splicing only upon hydration and activation of development [15], as well as intron retention in the transcripts of activated macrophage [16]. Incomplete and unspliced transcripts are usually degraded [17], so delayed splicing must somehow also involve RNA stabilization. In some cases, incomplete RNA processing results in retention of transcripts on chromatin, but the mechanism of retention and release is unknown [16,18–20].

If introns just cause delays, why bother having them? It is well known that the presence of introns in genes enhances their transcription [21,22]. Possible interpretations are that co-transcriptional processes feedback to the promoter or change the processivity of Pol II. In plants, evidence that sequences harbored within introns affect transcription elongation suggests that DNA- or RNA-based mechanisms could operate through melting temperature/secondary structure and/or through recruitment of specific factors [23]. Another recent study revisited this phenomenon and showed that introns and splicing activity influence promoter-proximal chromatin profiles, Pol II occupancy, and overall transcriptional output [24]. Consistent with these observations, intron-containing genes also have higher levels of H3K36me3, which is deposited by transcription-dependent mechanisms [25]. Strikingly, short first exons were shown to have more defined peaks of activating histone marks closer to the transcription start site (TSS), enhancing transcription accuracy and output [24]. Genes with long first exons are less well-expressed and display reduced accuracy at the TSS. The link between chromatin marks and gene-architecture is also evident at internal exons, which are preferentially bound by nucleosomes [10,26]. Interestingly, gene-specific elongation rates (see above) are related to these features [3,4]. Thus, intron/exon content and length are parameters that regulate transcriptional output and can be selected for in evolution.

Recent findings indicate that the requirement for specific gene architectures differ according to the cellular and developmental context. For example, genes involved in rapid biological responses may tend to be intron-poor, as they have to be quickly and efficiently induced [27]. In tissue patterning and artificial model systems, the presence of long introns serves as a timing mechanism for biological signals in feedback regulatory networks [28–30]. As transcription takes time, maturation of a gene product will be delayed if long introns are present in comparison to shorter genes, a principle termed intron delay [31–35]. Further, some introns harbor non-coding RNAs such as miRNAs or snoRNAs, whose processing from introns can speed up or slow down the rate of expression of the host gene [36–38].

The cell cycle is also a factor, since transcription and splicing are generally inhibited during mitosis [39–41]. The fastest cell cycles occur in rapidly developing early embryos: 8 minutes/cycle in the fruitfly *Drosophila melanogaster*, 15 minutes in the zebrafish *Danio rerio* and 30 minutes in the frog *Xenopus laevis* [42]. Recent high-throughput transcriptomic studies have shown that the earliest transcribed genes are short and often intronless, which should facilitate expression under the constraint of very short cell cycles [43–45]. Guilgur and co-workers further reported that highly efficient splicing is required during early fly embryogenesis [46], echoing the finding that inhibition of efficient assembly of spliceosome components is lethal during the rapid early zebrafish development [47]. These findings indicate that cell-cycle constraints influence the evolution of gene-architecture.

## Early zygotic genes are short, intron-poor and require efficient splicing

**Figure 2:**
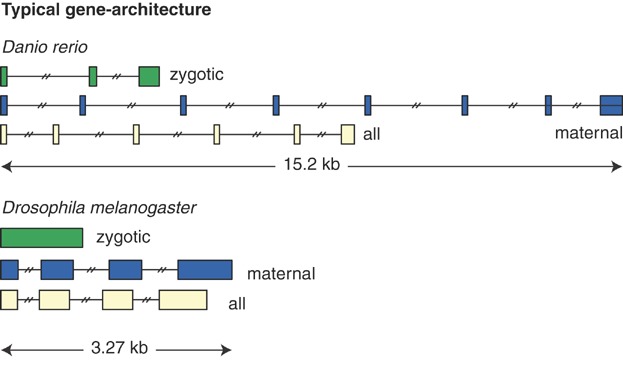
Distinct gene architectural features of maternal and zygotic genes. Stick diagram of typical gene architecture for zygotic, maternal, and all annotated transcripts in zebrafish (upper panel) and fly (lower panel). Drawn to scale is the median length of the genes and the first and last exons. For internal exons, the population median for all exons per transcript is drawn. Introns are not to scale; median numbers of introns are shown. Data are from Heyn et al. 2014 [44].

Recently Guilgur et al. [46] showed that efficient splicing is required during rapid early *Drosophila melanogaster* embryogenesis. The authors characterized two mutant alleles of the gene *fandango*, which encodes a component of the spliceosome complex NTC/Prp19, and measured splicing defects in maternally deposited and zygotically transcribed genes in *fandango* mutant embryos by RT-PCR and RNAseq. The results confirmed that NTC/Prp19 complexes are required for efficient spliceosome activity. Interestingly, while maternally deposited transcripts from unfertilized eggs and ovaries showed normal splicing patterns, early transcribed intron-containing zygotic transcripts showed a high degree of intron retention. Intriguingly, ectopic maternal expression of a zygotic gene rescued the splicing defect observed, indicating that not sequence but developmental context caused intron retention. Consistent with this, the authors observed a higher degree of intron retention for zygotic transcripts when compared to maternal transcripts even in wild-type embryos. A plausible alternative hypothesis is that unspliced maternal transcripts were degraded during the time it takes to produce a mature oocyte (12 days); in contrast, zygotic RNAs represent transcription and processing products from a time window of minutes to hours, which may be too short for unspliced RNAs to be fully degraded. Overall, this study suggests that the short syncytial cycles in early *Drosophila* embryogenesis favor transcripts with a simple gene-architecture consisting of short, intron-poor transcripts (Fig. 2). This conclusion is consistent with the conclusions of two other studies in mosquito and zebrafish embryos [43,44] as well as an independent analysis of the *Drosophila* early embryonic transcriptome [45].

The conserved trend toward short, intron-poor transcripts among the first zygotically expressed genes extends to the mouse [44], even though the first cell cycles are longer than in fly, frog, or zebrafish embryos. But with cell cycle lengths of 14–20 hours, there is still less time available for transcription than in most cells [48]. Another reason for keeping early zygotic genes short, especially the ones with potent patterning activities, may be the necessity to activate them or shut them down quickly. A phenomenon known as *repression lag* has been described for targets of transcriptional repressor *snail* in early *Drosophila* embryo [49]. The targets continue to be transcribed even after transcription initiation has been blocked by *snail* simply because the RNA polymerases present on the gene finish the job. The extent of the lag is then obviously dependent on the size of the gene, i.e. short genes can be shut-down most abruptly.

It is likely that the shortness of the first transcribed genes is also important for the coordination of transcription and replication, as the cell cycle during early development in fish, frog, and fly consists only of M and S phases. G phases are only gradually induced [50,51], therefore a considerable amount of transcription must take place during S phase and short gene length will aid the temporal separation of transcription and replication. This prediction is borne out by the histone genes, which are intronless and cluster in the genome [52,53]. Transcription of canonical histone genes is upregulated during S phase [53], when replication takes place and their special organization in the genome is thought to promote fast expression, likely to avoid interference with the replication machinery. Indeed histone transcripts are among the genes transcribed during short zygotic cell cycles in early development and are relatively short, intronless genes [44,54]. In contrast, it was shown that long genes are prone to DNA breakage, as transcription takes too long to separate it in time from replication [55]. Other rapid developmental periods are the very short cell cycles during gastrulation in rodents with only 3–3.5 hours in the primitive streak of rats [56] or the rapid cell cycles of neural progenitors during early murine neurogenesis [57]. Based on the observations described above, it is likely that genes transcribed in these fast cycling cells will exhibit a similar constraint in gene-architecture and the interplay between transcription and replication.

Importantly, absence of introns or gene length alone does *not* predict gene expression during fast cell cycles. First, not *all* short genes are expressed during early embryogenesis and, second, introns in some of the expressed short genes might feedback positively to facilitate rapid transcription [24,44]. For rapid expression, the best genes are short with a few introns and a short first exon. This, in fact, describes the architecture of immediate early genes, such as *FOS* and *MYC*, whereby transcripts robustly appear and disappear within 3 hours in cycling cells with much longer interphases and even in post-mitotic cells like neurons [58]. It is important to realize that the cell cycle constraints on gene length can be overcome by various means, so not all genes in the genome will tend toward shortness. For instance, genes acting in early *Drosophila* embryos are functionally pleiotropic and the forms expressed later in development (e.g. in neurons) often sport very long 3′ UTRs [59,60]. Alternative polyadenylation (APA) site selection is emerging as a mechanism for generating short and long alternative 3′ UTRs [61]; APA in turn can redefine gene length and introduce delays or short cuts, similar to introns.

## Long genes with introns delay expression

In contrast to periods in which genes must be quickly expressed, the proper function of the vertebrate segmentation clock seems to depend on delays introduced by the presence of introns. The segmentation clock is a genetic oscillator which gives rise to somites during embryo development [62]. Mathematical modeling predicts that the oscillations depend on a negative feedback loop with an appropriate delay in protein expression, which could be a transcriptional delay introduced by long introns or a processing delay, e.g. splicing and mRNA export [62]. Excitingly, splicing seems to delay the expression of the oscillator gene Hes7 [63] and deletion of all or two introns of Hes7 in mouse embryos abolishes or shortens the oscillations leading to altered somite formation [64,65]. Thus, the hypothesis of intron-delay holds true in vivo.

Genes transcribed during oogenesis and deposited into the egg are large and harbor more introns than zygotic genes [44]. As cell cycles during oogenesis are longer, there is enough time to produce large transcripts, harboring many introns. Alike, some of the longest human genes such as DLG2 (2.17 Mb) or NRXN3 (1.46 Mb) [66] are expressed in neurons, which are terminally differentiated cells and therefore cell cycle constraints on transcription unit size do not exist. Large genes with multiple introns can produce very complex proteins with many different domains that fulfill complex functions. Comparison of six *Drosophila* species shows that expression of transcripts with long introns is delayed during embryogenesis in all species, indicating that intron delay plays an important role in regulation of gene expression during embryogenesis [45]. Simultaneously, the presence of introns offers the potential for regulatory functions such as alternative splicing to create functionally different proteins from a single gene.

Alternative mechanisms how introns can delay expression of a certain transcript are intron retention and post-transcriptional splicing, which regulate RNA abundance as well as protein translation in certain cellular contexts. Early spermatogenesis in the fern *Marsilea vestita* is transcriptionally quiescent, analogous to early embryogenesis. Development of the gametophyte depends on stored RNA, whose protein products are needed at different time points. In the absence of transcription, protein production must be controlled post-transcriptionally. Regulation is achieved, at least in part, by intron retention in the stored RNA and subsequent splicing of retained introns to allow protein production [15]. Importantly, the average length of the retained introns in fern spermatogenesis is 179 bp and therefore these introns are distinct from the very long introns discussed above. It is not known if this mechanism of introducing a delay in gene expression by delaying splicing generalizes to many other systems, besides delays in splicing documented in activated white blood cells (see above and Refs. [12,16]). However, cytoplasmic splicing of pre-mRNAs stored in anucleate platelets provides a compelling example in vertebrates [67].

## From cells to organisms: The evolutionary consequences of selection for short genes

The notion that cellular constraints might influence the evolution of gene- and genome-level architectures is not new. For example, Cavalier–Smith [68,69] proposed that the polycistronic structure of prokaryotic mRNAs is a consequence of the longer time required to replicate DNA than to duplicate the cell. This cellular constraint means that growing populations of prokaryotic cells initiate several rounds of DNA replication in a staggered fashion to ensure that duplication of the genome does not dramatically slow down the rate of cell growth. However, since there is a single origin of replication (*ori*) in the bacterial genome, genes that are located close to the *ori* are likely to be present in multiple copies in individual bacterial cells leading to the location of highly expressed genes close to the *ori*, and weakly expressed genes close to the terminus [70]. This gene dosage effect might in turn favor the organization of genes involved in closely related functions into polycistronic mRNAs to ensure an equality in the levels of their protein products. The presence of multiple origins of replication in eukaryotes, together with more complex translational regulation, could explain both the absence of this unit of genome organization and the broad chromosomal distribution of genes involved in related functions that is exhibited by this group of organisms.

**Figure 3:**
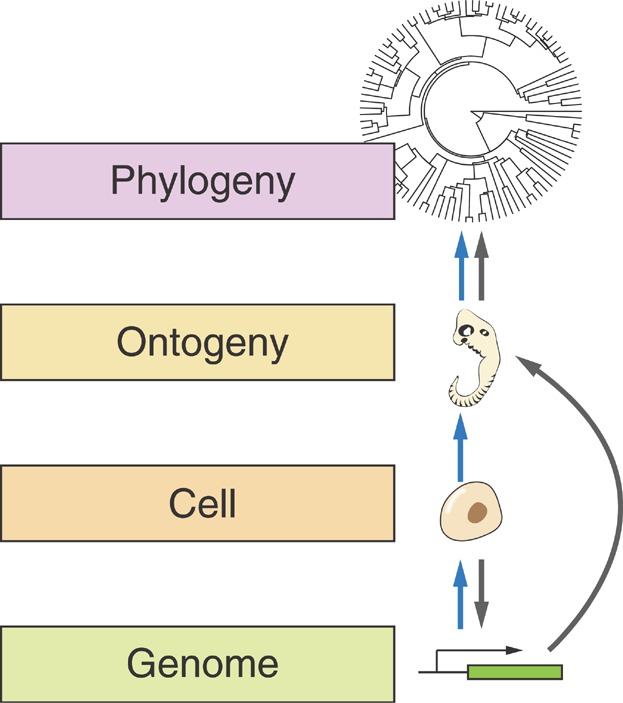
Causative relationships between different levels of biological organization. Blue arrows indicate relationships that propagate from low to high via all the intermediate levels. In contrast, gray arrows indicate potentially new relationships in which fast cell cycles constrain genes to be short, which in turn increases the fraction of young genes that are expressed at specific stages of ontogeny. This alternative path illustrates how causative effects can take different routes as a consequence of the impact of cellular constraints on genomic architectures.

We might be tempted to ask whether such cellular constraints have evolutionary consequences above the level of genomic organization. The discovery that the rapid cell cycles of early development constrain the length of genes expressed during this period can be embedded in the larger context by noting that short genes also tend to be evolutionarily young [7,52,71]. In accordance with this finding, early zygotic genes tend to be evolutionarily younger than genes expressed at other stages of development in both zebrafish and *Drosophila* [44]. A higher propensity for the expression of young genes suggests that this period of development may be inherently more evolvable, a proposal that is consistent with the hourglass model in which greater evolutionary divergence is predicted in the earliest periods of development relative to middle periods [72,73]. More generally, the ability to connect specific aspects of cellular dynamics with gross patterns of evolution and biodiversity is quite remarkable, and hints at the existence of many more undiscovered links between these two, often considered disparate, levels of biological organization (Fig. 3).

## Conclusion

Here we have elaborated current knowledge of the time needed for the gene expression machinery to act on the genome and how that compares with time intervals experienced by cells and tissues. Our argument that cellular constraints shape the transcriptome may well extend to other features of cellular function. In particular, future investigations are needed to understand in more detail the connection between cell cycle constraints and gene expression. For example, how Pol II elongation rates vary during different phases of the cell cycle, development and in different cell types is unknown. How prevalent is Pol II pausing? Is intron retention a widespread mechanism for regulating gene expression? Combining new sequencing technologies with metabolic labeling will allow researchers to pinpoint actively transcribed genes in other rapidly cycling or terminally differentiated cells, providing the basis for connecting gene architecture with cell cycle dynamics. In addition, single molecule fluorescence in situ hybridization (FISH) make single-cell analysis in the context of a whole organ or developing animal possible. As early cellular processes are highly dynamic, live imaging of transcriptional activity may be necessary to study the interplay between the cell cycle progression and molecular processing in the nucleus [74]. These findings will provide insight into the existence of connections between the lowest levels of biological organization and the evolutionary forces that shape major patterns of biodiversity.
